# Surface-Plasmon-Resonance-Based Optical Fiber Curvature Sensor with Temperature Compensation by Means of Dual Modulation Method

**DOI:** 10.3390/s18082608

**Published:** 2018-08-09

**Authors:** Yudong Su, Yong Wei, Yonghui Zhang, Chunlan Liu, Xiangfei Nie, Zongda Zhu, Lu Liu

**Affiliations:** 1 Chongqing Municipal Key Laboratory of Intelligent Information Processing and Control of Institutions of Higher Education, Chongqing Three Gorges University, Wanzhou, Chongqing 404100, China; 20160009@sanxiau.edu.cn; 2 College of Electronic & Information Engineering, Chongqing Three Gorges University, Wanzhou, Chongqing 404100, China; 20160012@sanxiau.edu.cn; 3 Basic Medicine Department, Chongqing Three Gorges Medical College, Wanzhou, Chongqing 404100, China; pharmacology-zyh@njucm.edu.cn; 4 Chongqing Engineering Research Center of Internet of Things and Intelligent Control Technology, Chongqing Three Gorges University, Wanzhou, Chongqing 404100, China; guangxianchuangan@njust.edu.cn; 5 National Key Laboratory of Science and Technology on Tunable Laser, Harbin Institute of Technology, Harbin 150001, China; 18b921014@stu.hit.edu.cn; 6 Department of Physics, Harbin Institute of Technology, Harbin 150001, China; 18b311002@stu.hit.edu.cn

**Keywords:** fiber optics sensors, surface plasmon resonance, curvature measurement, dual-modulation method, temperature compensation

## Abstract

Curvature measurement plays an important role in many fields. Aiming to overcome shortcomings of the existing optical fiber curvature sensors, such as complicated structure and difficulty in eliminating temperature noise, we proposed and demonstrated a simple optical fiber curvature sensor based on surface plasmon resonance. By etching cladding of the step-index multimode fiber and plating gold film on the bare core, the typical Kretschmann configuration is implemented on fiber, which is used as the bending-sensitive region. With increases in the curvature of the optical fiber, the resonance wavelength of the SPR (Surface Plasmon Resonance) dip linear red-shifts while the transmittance decreases linearly. In the curvature range between 0 and 9.17 m^−1^, the wavelength sensitivity reached 1.50 nm/m^−1^ and the intensity sensitivity reached −3.66%/m^−1^. In addition, with increases in the ambient temperature, the resonance wavelength of the SPR dips linearly blueshifts while the transmittance increases linearly. In the temperature range between 20 and 60 °C, the wavelength sensitivity is −0.255 nm/°C and the intensity sensitivity is 0.099%/°C. The sensing matrix is built up by combining the aforementioned four sensitivities. By means of the dual modulation method, the cross-interference caused by temperature change is eliminated. Additionally, simultaneous measurement of curvature and temperature is realized.

## 1. Introduction

Curvature sensors have a wide number of applications, including structural health monitoring in civil engineering, intelligent artificial limbs, soft robotic configurations, and in mechanical engineering [1,2,3]. In the last decade, the optical fiber sensor has developed rapidly because of its superior capabilities, as compared with traditional electronic sensors, including its small size, light weight, instant response, anti-electromagnetic interference, attribute of being chemically inert, long distance transmission and on-line monitoring, etc. So far, various optical fiber curvature sensors have been proposed based on fiber Bragg gratings (FBGs) [4,5,6], long-period fiber gratings (LPFGs) [7,8,9], optical fiber Mach-Zehnder interferometers (OFMZIs) [10,11], singlemode-multimode-singlemode (SMS) optical fiber structure [12,13], and so on. However, the FBGs and LPFGs are sensitive to ambient temperature variation, which may induce the cross-interference and decrease in the accuracy of the sensor. The OFMZIs and SMS structures are usually based on microstructure fibers such as the multicore fiber and photonic crystal fiber, which leads to a complex manufacturing process and high cost. Although these curvature sensors usually have a high sensitivity, they are not favorable for implementation due to these shortcomings, as described above.

Surface plasmon resonance (SPR) optical fiber sensors have attracted much attention for decades, due to their advantages of high sensitivity, high accuracy, both a simple and multifarious structure, and low cost. They have been applied for refractive index detection of chemical and biological analytes [14,15,16,17]. Compared to other optical fiber sensors based on FBG, LPFG, optical fiber interferometer (OFI) and so on, the SPR fiber sensors have more excellent performances such as ultrahigh sensitivity up to several microns per RIU. This means the SPR fiber sensors have a smaller detection limitation under the same wavelength resolution of detector [18]. However, in the fields of the physical parameters measurement, such as curvature and displacement measurement, the applications of the SPR fiber sensor are still scarce because there are some barriers to control the propagation angle of the light beam in the fiber and the intensity of the evanescent wave. In recent years, some studies have proved, by theoretical and experimental methods, that bending optical fibers into a u-bent can enhance the evanescent wave intensity, thereby enhancing the depth of the SPR valley [19,20,21,22]. These sensors are only used for refractive index measurements, however, they perhaps provided an earlier idea: SPR fiber sensors can measure the curvature of the fiber through intensity detection. Later, some research on the bending effect of the SPR fiber sensor began to emerge. Tagaki et al [23]. proposed a SPR sensor based on a curved hetero-core fiber. The experiment proved that the SPR loss spectrum peak increases about 3.8 dB and the resonance wavelength red-shifts 9 nm as the curvature increases from 0 to 22.6 m^−1^, while the sensitivity was low. Napiorkowski et al. [24]. and Dash et al. [25] researched the bending effect of the SPR sensor, based on the photonic crystal fiber, by simulation, and the results proved the similar change law as the literature above. However, due to the high complexity and difficult fabrication process of the structure, they did not experimentally implement the sensor. A similar experiment was also carried out later by Gasior et al. [26]. However, in this study, only the intensity change was regular while the wavelength change was chaotic, which made the wavelength modulation not available. Thus, this sensor could not eliminate the interference of the refractive index or temperature. All in all, although this literature has studied the effect of bending on the resonant valley of SPR fiber sensors, the sensors are not really used to measure curvature but are used to optimize refractive index sensing. In addition, the studies also did not take the ambient temperature into consideration.

In this paper, we proposed and demonstrated a simple optical fiber curvature sensor based on SPR. To the best of our knowledge, it is the first time that the SPR sensor can simultaneously measure curvature and temperature. We etched the cladding of the step-index multimode fiber and fabricated the Kretschmann configuration on the bare fiber core, which is used as bending-sensitive region. With the increasing curvature of the optical fiber, the resonance angle decreased gradually, which lead to a red-shift of the resonance wavelength of the SPR dip; meanwhile, the intensity of evanescent wave also increased, which lead to a decline of the transmittance of the SPR dip. Thus, we were able to measure the curvature by wavelength demodulation or intensity demodulation. In addition, we researched the effect of the ambient temperature on the proposed sensor. With increases in temperature, the resonance wavelength blue-shifts while the transmittance decreases. Thus, we were able to measure temperature by wavelength demodulation or intensity demodulation. Combing these four sensitivities, we built up the sensing matrix, through which we could compensate the temperature interference. In addition, we also found that the sensitivity can be enhanced by using the optical fiber with a smaller numerical aperture (NA). The proposed optical fiber curvature sensor has the advantages of simple structure and easy fabrication process, but, most of all, it can simultaneously measure curvature and temperature by using only one SPR sensing region.

## 2. Sensor Structure

**Figure 1:**
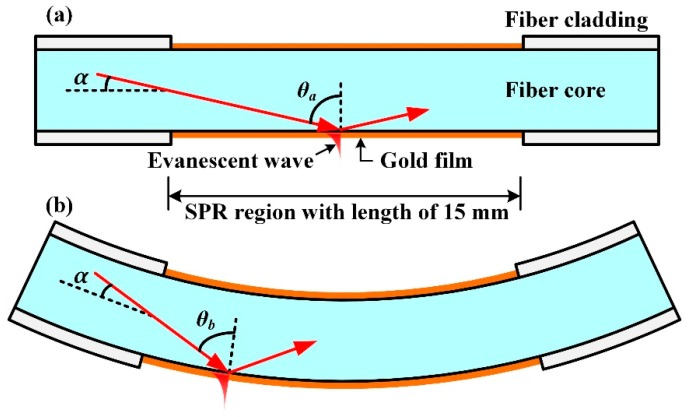
The sketch diagram of the fiber SPR (Surface Plasmon Resonance) bending sensor. (**a**) SPR region in the straight case; (**b**) SPR region in the bending case.

Figure 1 shows the sketch diagram of the optical fiber bending sensor. We fabricated the SPR region with a length of 15 mm in the middle of a step-index multimode fiber. The core diameter of the fiber is 105 μm, the cladding diameter is 125 μm and NA is 0.22. The fiber cladding in the SPR region was removed by means of chemical etching method so that evanescent wave could penetrate out from the bare fiber core. A layer of gold film is plated on the bare fiber core for forming the Kretschmann configuration and exciting the SPR phenomenon. The light beam propagating in the multimode fiber has numerous modes; in other words, the incident angle is divergent between critical angle and 90°. However, according to Iga [27], only a very narrow incident angle range plays a significant role for SPR excitation. To simplify the analysis, we assumed the included angle between the useful light beams and the fiber axis was a single value *α*. When the fiber was in the straight case, as shown in Figure 1a, the light beam undergoes total internal reflection with incident angle *θ*_a_ = 90° − *α* on the surface of fiber core while exciting the SPR phenomenon. When the fiber is in the bending case, as shown in Figure 1b, the incident angle *θ*_b_ was slightly smaller than *θ*_a_, which caused the resonance wavelength red-shift slightly. In addition, because the incident angle was closer to the critical angle, the evanescent wave intensity enhanced, which lead to a deeper SPR dip.

In order to ascertain the optimal thickness of the gold film, we simulated the SPR transmitted spectrum using the Fresnel formula. The transmittance can be modelled by:

(1) $$R={\left|\frac{r_{01}+r_{12}e^{2ik_{z}h}}{1+r_{01}\cdot r_{12}e^{2ik_{z}h}}\right|}^{2}$$

where the subscripts 0, 1, and 2 stand for the fiber core, gold film, and ambient medium, respectively. *h* is the gold film thickness, *r*_01_ is the reflectivity at the interface between fiber core and gold film, *r*_12_ is the reflectivity at the interface between gold film and external medium, and *k_z_* is the *z* component of the wave vector in the gold film. According to the Fresnel formula, the reflectivities can be modelled by:

(2) $$r_{ik}=\frac{{\left(\varepsilon_{i}-\varepsilon_{0}\sin^{2}\theta \right)}^{0.5}/\varepsilon_{i}-{\left(\varepsilon_{k}-\varepsilon_{0}\sin^{2}\theta \right)}^{0.5}/\varepsilon_{k}}{{\left(\varepsilon_{i}-\varepsilon_{0}\sin^{2}\theta \right)}^{0.5}/\varepsilon_{i}+{\left(\varepsilon_{k}-\varepsilon_{0}\sin^{2}\theta \right)}^{0.5}/\varepsilon_{k}}$$

(3) $$k_{z}=\frac{2\pi }{\lambda }{\left(\varepsilon_{1}-\varepsilon_{0}\sin^{2}\theta \right)}^{0.5}$$

where the subscripts *i*, *k =* 0, 1, 2; and *θ* is the incident angle.

**Figure 2:**
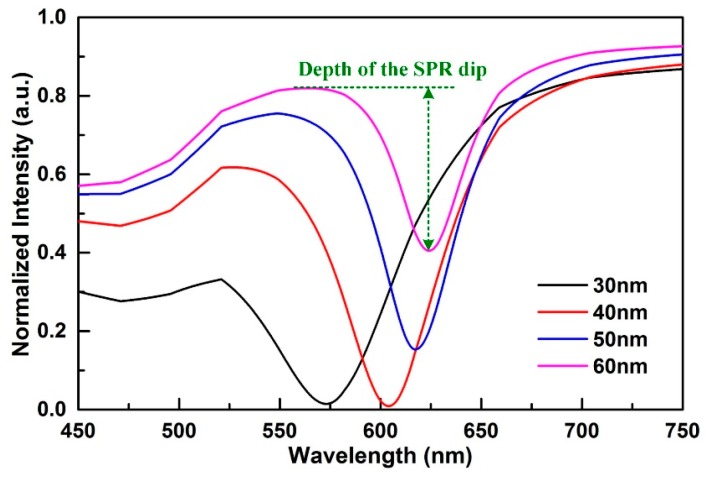
The transmitted spectrum under different gold film thicknesses.

Figure 2 provides the simulation results with gold thicknesses of 30, 40, 50 and 60 nm, respectively, when the fiber was in the straight case. The simulated conditions were a: Refractive index of the fiber core of 1.465, refractive index of the ambient medium of 1.333 (water), and an incident angle, *θ*_a_, of 82°. When the gold film thickness was 30 nm, the depth of the SPR dip was shallow. When the gold film thickness increased to 40 and 50 nm, the depth of the SPR dip increased significantly. When the gold film thickness increased to 60 nm, the depth of the SPR dip became shallow again. Thus, the optimal thickness of the gold film for exciting SPR was 40 to 50 nm and, here, we chose the 45 nm gold film for the experiment.

## 3. Experimental Preparation

### 3.1. Fabrication of the Bending Sensor

The fabrication processes for the bending sensor were as follows. Firstly, we took an optical fiber with a length of 2 m and removed the coating layer with a length of 15 mm in its middle position as the SPR region. The cladding and core of the optical fiber were both silica glass materials that were difficult to separate, so we used the HF solution with a concentration of 40% to etch the fiber cladding and expose the fiber core. To avoid the HF solution diffusion, caused by the capillary phenomenon, which would cause corrosion of the other regions of the optical fiber, we used a paraffin to block both ends of the SPR region. The SPR region is immersed in the HF solution, and, after 5 min, the diameter of the SPR region decreased to 100 μm, which meant the fiber cladding was totally removed. Then, we washed the SPR region with a mass of water and dried it. Finally, we plated the gold film on the surface of the SPR region by using a plasma sputtering apparatus (JS-1600; Beijing HTCY Technology, Beijing, China). To ensure the gold film surface was smooth with a consistent thickness, we installed a motor in the vacuum chamber of the plasma sputtering apparatus, which drove the fiber rotating at a constant speed during gold film coating.

**Figure 3:**
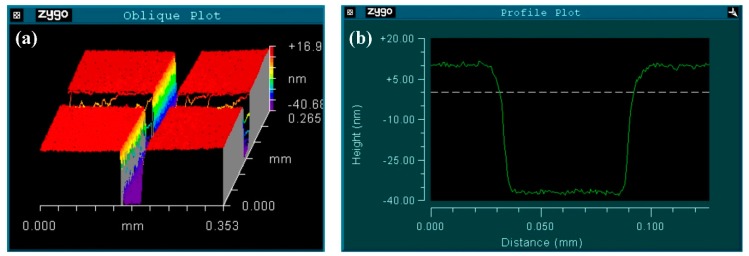
(**a**) Three-dimensional testing result for the gold coating with a groove; (**b**) test result for the groove depth, which is equal to the gold coating thickness.

We employed a three-dimensional morphology analyzer (New View7200, Zygo, Middlefield, CT, USA) to evaluate the gold coating quality. The gold film was scratched in a cross-shape groove. The flatness and the depth of the gold coating were observed (see Figure 3a,b). The depth of the groove indicated the thickness of the gold coating, which was 45 nm.

### 3.2. Experimental Setup

**Figure 4:**
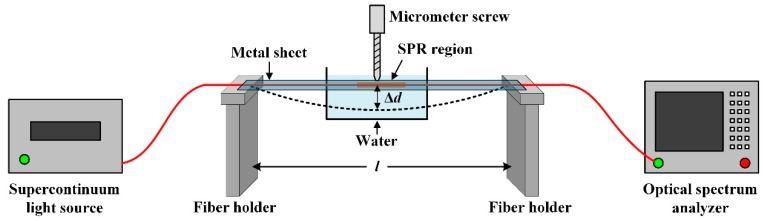
The sketch diagram of the fiber SPR bending sensor experiment system.

The experimental setup is shown in Figure 4. The optical fiber containing the SPR region was fixed on a metal sheet, which was placed on two fiber holders. A micrometer screw installed in the middle position of two fiber holders was used to bend the metal sheet together with the optical fiber. Due to the elasticity of the metal sheet, the optical fiber between the two fiber holders formed a perfect arc with curvature *k*. To excite the SPR phenomenon, we immersed the SPR region into water (the refractive index of the ambient medium was 1.333). The distance between the two fiber holders was *l =* 12 cm. The accuracy and travel of the micrometer screw were 0.01 and 25 mm respectively. We defined the maximum offset away from the straight line as Δ*d*. By calculation, the functional relationship between *k* and Δ*d* can be expressed as:

(4) $$k=\frac{8\Delta d}{l^{2}+4{\left(\Delta d\right)}^{2}}$$

The laser from the supercontinuum light source (SuperK compact, NKT Photonics, Birkerød, Denmark), whose spectrum range was 450 to 2400 nm, was launched into the optical fiber, which then excited the SPR phenomenon in the SPR region. The transmitted beams were then received by an optical spectrum analyzer (AQ6373, Yokogawa, Tokyo, Japan) for which the detection range was 350 to 1200 nm and the wavelength resolution was 0.02 nm.

## 4. Results and Discussion

### 4.1. Bending Test

**Figure 5:**
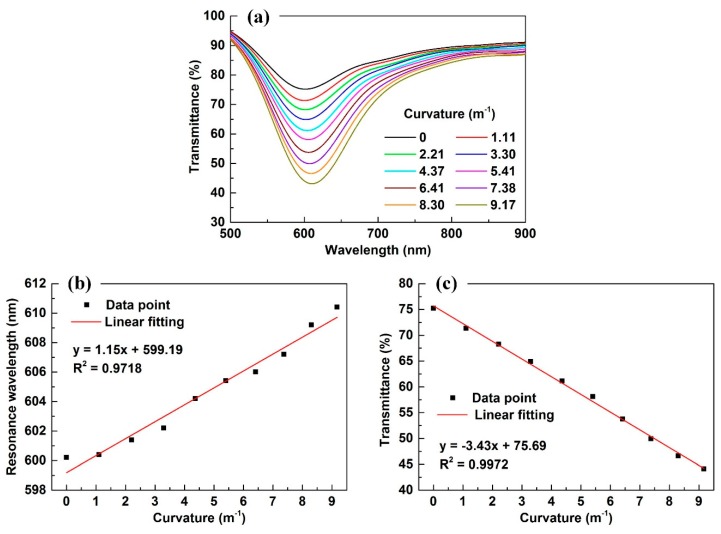
(**a**) The transmitted spectra under a series of curvatures; (**b**) the resonance wavelength as a function of curvature; (**c**) the transmittance as the function of curvature.

At ambient temperature of 20 °C, we collected transmitted spectra from a displacement value, Δ*d,* that increased from 0 to 18 mm with the interval of 2 mm, which corresponded to a curvature of 0 to 9.17 m^−1^. The spectrum, when the optical fiber is in the straight case and in the air surrounding, is used as a reference to normalize all the above obtained spectra. In addition, the spectrum of the light source has large fluctuation and noise, which will make the resonance wavelength difficult to identify. For solving this issue, we used the smooth function from MATLAB to obtain the smooth spectra; the number of smooth function is 300. The experimental results are shown in Figure 5. As shown in Figure 5a, when the optical fiber is in the straight case, i.e., curvature is 0 m^−1^, the minimum transmittance of the SPR dip is 75.2%, and the resonance wavelength is 600.2 nm. As the curvature increased, the minimum transmittance of the SPR dip decreased while the resonance wavelength red-shifts slightly. When the curvature increased to 9.17 m^−1^, the minimum transmittance decreased to 44.1% and the resonance wavelength red-shifts to 610.2 nm.

Figure 5b provides the resonance wavelength as the function of curvature. Using linear fitting, the functional relationship between the resonance wavelength (*λ*/nm) and curvature (*k*/m^−1^) can be expressed as:

(5) λ=(1.15±0.06)k+(599.19±0.4)

Figure 5c provides the transmittance as the function of curvature. Using linear fitting, the functional relationship between transmittance (*I*/%) and curvature (*k*/m^−1^) can be expressed as:

(6) I=(−3.43±0.09)k+(75.69±0.5)

The experimental results show that both the transmittance and resonance wavelength are sensitive to the curvature of the optical fiber bending sensor. The intensity sensitivity is −3.43%/m^−1^ and the wavelength sensitivity is 1.15 nm/m^−1^.

### 4.2. Effect of the Optical Fiber NA on Sensitivity

**Figure 6:**
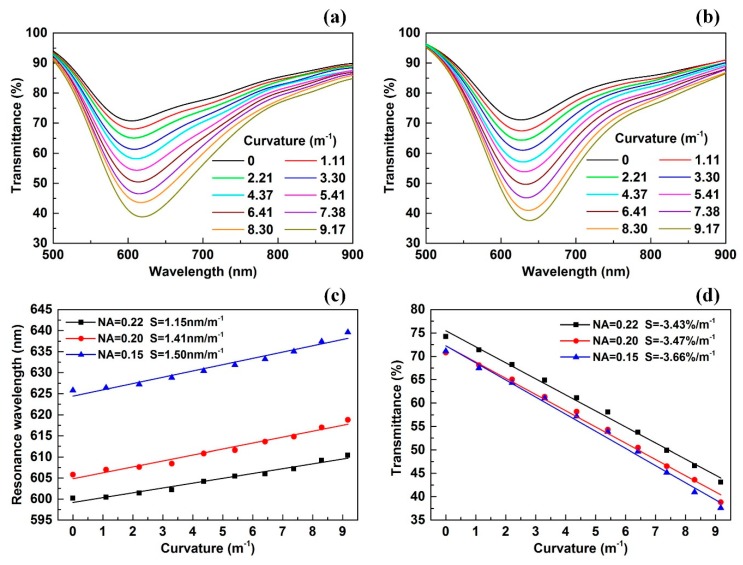
(**a**) The transmitted spectrum changing with curvature when fiber NA = 0.20; (**b**) the transmitted spectrum changing with curvature when fiber NA = 0.15; (**c**) comparison of the resonance wavelength variation under different fiber NAs; (**d**) comparison of the transmittance variation under different fiber NAs.

We also researched the effect of the optical fiber NA on the sensing performance. The optical fiber with the same cladding and core diameters, but with different NA, was used to fabricate the new bending sensor. The fabrication process was the same as before. Figure 6a shows the transmitted spectrum variation when the optical fiber NA = 0.20 and Figure 6b shows the transmitted spectrum variation when the optical fiber NA = 0.15. The spectrum variation was basically the same as the previous experimental result. Figure 6c shows the comparison between the resonance wavelength variations under different optical fiber NAs. When the optical fiber NA = 0.22, the resonance wavelength increases from 600.2 to 610.4 nm, and the wavelength sensitivity is 1.15 nm/m^−1^. When the optical fiber NA = 0.20, the resonance wavelength increased from 605.8 to 618.8 nm, and the wavelength sensitivity was 1.41 nm/m^−1^. When the optical fiber NA = 0.15, the resonance wavelength increased from 626.4 to 639.8 nm, and the wavelength sensitivity was 1.50 nm/m^−1^. According to Figure 6c, as the optical fiber NA decreased, the resonance wavelength range red-shifts, meanwhile, the wavelength sensitivity slightly increases. Figure 6d shows the comparison of the transmittance variation under different optical fiber NAs. When the optical fiber NA = 0.22, the transmittance decreased from 75.2 to 44.1%, and the intensity sensitivity was −3.43%/m^−1^. When the optical fiber NA = 0.20, the transmittance decreased from 70.8 to 38.8%, and the intensity sensitivity was −3.47%/m^−1^. When the optical fiber NA = 0.15, the transmittance decreased from 71.1 to 37.6%, and the intensity sensitivity was −3.66%/m^−1^. According to Figure 6d, as the optical fiber NA decreases, the transmittance decreased slightly, while the intensity sensitivity also slightly increased.

On the other hand, the figure of merit (FOM) and the detection limitation (DL) were also important parameters to evaluate the SPR sensor. The higher the FOM, the better the sensor. The smaller the DL, the better the sensor. Refer to [28], the FOM can be expressed as FOM = S/FWHM, and the DL can be expressed as DL = R_D_/S, where S is wavelength sensitivity, FWHM is full width at half maximum obtained by single-peak Gaussian fitting, and R_D_ is the wavelength resolution of the detector (for our experiments, R_D_ = 0.2 nm). Table 1 shows the comparison of parameters between three sensors under wavelength modulation. When there were decreases in the NA of fiber, the FOM increased and the DL diminished. It is noteworthy that the DL is a theoretical value, indeed, it may be affected by the noise of the light source. If the spectrum of the light source is sufficiently stable and smooth, the actual DL can reach the theoretical value.

### 4.3. Effect of the Temperature on Sensing Results

**Figure 7:**
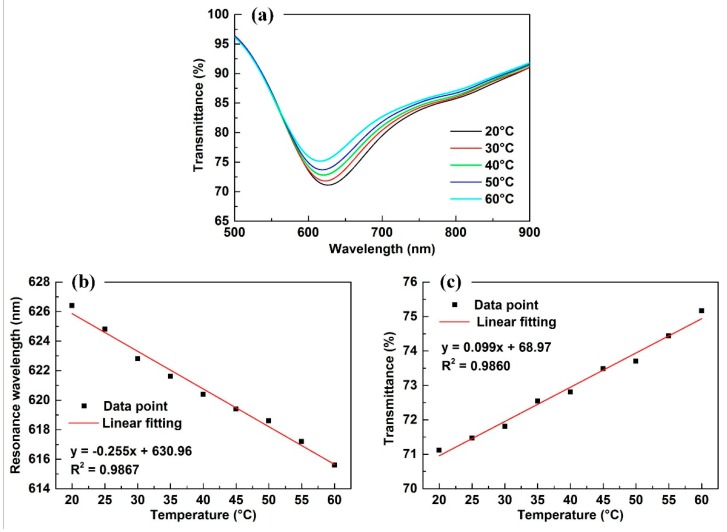
(**a**) The transmitted spectra under a series of temperatures; (**b**) the resonance wavelength as the function of temperature; (**c**) the transmittance as the function of temperature.

The SPR response was sensitive to the ambient temperature variation, so we needed to research the effect of the ambient temperature on the transmission spectrum. Under the condition of fiber NA = 0.15 and in the straight case, we changed the ambient temperature by using a heater and collected the corresponding transmission spectrum. Figure 7a shows the transmission spectra under a temperature of 20, 30, 40, 50 and 60 °C. When the temperature increased from 20 to 60 °C, the minimum transmittance of the SPR dip increased slightly from 71.1% to 75.2%, and the resonance wavelength blue shifted slightly from 626.4 to 615.6 nm.

Figure 7b provides the resonance wavelength as the function of temperature. Using linear fitting, the functional relationship between resonance wavelength (*λ*/nm) and temperature (*T*/°C) can be expressed as:

(7) λ=(−0.255±0.01)T+(630.96±0.4)

Figure 7c provides the transmittance as the function of temperature. By linear fitting, the functional relationship between transmittance (*I*/%) and temperature (*T*/°C) can be expressed as:

(8) I=(0.099±0.004)T+(68.97±0.2)

The experimental results show that both the transmittance and resonance wavelength were sensitive to the ambient temperature when the intensity sensitivity was 0.099%/°C and the wavelength sensitivity was −0.255 nm/°C.

Essentially, the resonance wavelength variation induced by the temperature was induced by the change in the refractive index of water. Referring to Reference [29], under a wavelength of 632.8 nm, the refractive index variation of water as a function of the temperature can be expressed as $n=-1.44\times 10^{-4}T+1.33524$, where the slope is the thermo-optic coefficient of water. So, we could calculate the refractive index sensitivity of the sensor is 1770 nm/RIU. Compared with the refractive index sensors that have similar structure [30], the refractive index sensitivity was almost at the same level.

### 4.4. Temperature Compensation of the Curvature Sensor

Because both the bending and ambient temperature lead to changes in the transmission spectrum, changes in the ambient temperature introduced severe cross-interference. However, what is interesting is that both the resonance wavelength and transmittance could be used to demodulate the curvature or ambient temperature. Thus, we can build up the sensing matrix by combining the above four sensitivities related to curvature and temperature. This method was effective in reference [31] and suitable for our sensor. Using the sensing matrix, we could compensate for the cross-interference caused by the temperature changes on the sensing results. Furthermore, we could simultaneously measure the curvature and ambient temperature too.

Changes in bending (Δ*k*/m^−1^) and ambient temperature (Δ*T*/°C) produced changes in the resonance wavelength (Δ*λ*/nm) and transmittance (Δ*I*/%), this relation could be mathematically expressed by the following set of equations:

(9) $$\left[\begin{array}{c} \Delta \lambda \\ \Delta I \end{array}\right]=\left[\begin{array}{cc} 1.50\pm 0.1 & -0.255\pm 0.01\\ -3.66\pm 0.1 & 0.099\pm 0.004 \end{array}\right]\left[\begin{array}{c} \Delta k\\ \Delta T \end{array}\right]$$

Finally, the sensing matrix could be then expressed as:

(10) $$\left[\begin{array}{c} \Delta k\\ \Delta T \end{array}\right]=\left[\begin{array}{cc} -0.1278\pm 0.018 & -0.3266\pm 0.020\\ -4.6937\pm 0.34 & -1.9062\pm 0.32 \end{array}\right]\left[\begin{array}{c} \Delta \lambda \\ \Delta I \end{array}\right]$$

By the sensing matrix, we could completely eliminate the cross-interference caused by changes in the ambient temperature. In addition, the curvature (*k*/m^−1^) could no longer be calculated separately from the resonance wavelength (*λ*/nm) or the transmittance (*I*/%), but was determined by them together. Here, we took the straight case, i.e., *k*_0_ = 0 m^−1^, *T*_0_ = 20 °C, *λ*_0_ = 624.43 nm and *I*_0_ = 72.25%, which were given by the fitting curve in Figure 6, as the initial status, so the bending sensing function can be expressed as:

(11) k=(−0.1278±0.018)(λ−624.43)+(−0.3266±0.020)(I−72.25)

In addition, if we take the fitting curve in Figure 7 as the initial status, the temperature sensing function can be expressed as:

(12) T=(−4.6937±0.34)(λ−630.96)+(−1.9062±0.32)(I−68.97)

Finally, by means of dual modulation method, the temperature compensation was realized. However, this method may still have some limitations. If the wavelength shift induced by the temperature change was similar in magnitude to the wavelength shift induced by the curvature change, while the intensity change induced by the temperature change was also similar to the intensity change induced by the curvature change, the inverse matrix of the Equation (9) would be unsolvable, so that we would not be able to get Equation (10). Once this case happened, it is feasible to replace the ambient liquid with a different thermo-optic coefficient or refractive index to solve this issue.

## 5. Conclusions

In summary, an optical fiber curvature sensor based on surface plasmon resonance was presented. By etching the cladding of the step-index multimode fiber and plating a 45 nm sensing gold film on the bare fiber core, the typical Kretschmann structure was implemented on fiber, which was used as bending-sensitive region. With increases in the curvature of the optical fiber, the resonance angle decreased gradually, which lead to red-shift in the resonance wavelength of the SPR dip; meanwhile, the intensity of evanescent wave also increased, which lead to a decline in the transmittance of the SPR dip. Thus, we could measure the curvature by wavelength demodulation or intensity demodulation. The sensitivity of the curvature sensor rose as the NA of the fiber decreased. When the NA was 0.15, the wavelength sensitivity reached 1.50 nm/m^−1^ and the intensity sensitivity reached −3.66%/m^−1^_,_ during the curvature range between 0 and 9.17 m^−1^.

The effect of the ambient temperature on the curvature sensor was also carried out. With increases in the ambient temperature, the resonance wavelength of the SPR dip blue-shifts, while the transmittance of the SPR dip increased. During the temperature range between 20 °C and 60 °C, the wavelength sensitivity was −0.255 nm/°C and the intensity sensitivity was 0.099%/°C. The curvature and temperature together affect the resonance wavelength and transmittance, which induced the cross-interference. In order to eliminate it, the sensing matrix associated with curvature and temperature was established. Finally, by means of dual modulation method, the temperature compensation was realized. Compared to the other types of optical fiber curvature sensors based on FBGs, LPFGs and OFIs, this optical fiber curvature sensor had the advantages of a simple structure and easy fabrication process, but, most of all, it could simultaneously measure the curvature and temperature using only one SPR sensing region.

## Figures and Tables

**Table 1:** Parameters comparison between three sensors using wavelength modulation.

Parameters	Sensor 1	Sensor 2	Sensor 3
NA of fiber	0.22	0.20	0.15
Sensitivity, nm/m^−1^	1.15	1.41	1.50
FWHM, nm	165	193	184
FOM, /m^−1^	6.97 × 10^−3^	7.31 × 10^−3^	8.35 × 10^−3^
DL, m^−1^	0.17	0.14	0.13
